# Evaluating Diagnostic Clarity: The Comparative Efficacy of BlueStain in Serous Effusion Cytology under the International System for Reporting Serous Fluid Cytopathology Reporting Framework

**DOI:** 10.3390/diagnostics14111074

**Published:** 2024-05-22

**Authors:** Paula Melo Alves, Maria Teresa Azevedo, Fernando Ferreira, Ebru Tastekin, Sule Canberk, Fernando C. Schmitt

**Affiliations:** 1 Pathology and Molecular Genetics, Institute of Biomedical Sciences Abel Salazar (ICBAS), University of Porto, 4050-313 Porto, Portugal; 2 Polytechnic Institute of Health of the North (IPSN), Cooperativa de Ensino Superior Politécnico e Universitário (CESPU), 4585-116 Paredes, Portugal; 3 UNIPRO—Oral Pathology and Rehabilitation Research Unit, University Institute of Health Sciences—(IUCS-CESPU), 4585-116 Paredes, Portugal; 4 Institute for Research and Innovation in Health (i3S), University of Porto, 4200-135 Porto, Portugal; 5 Department of Pathology, Medical Faculty, Trakya University, 22030 Edirne, Turkey; 6 Institute of Molecular Pathology and Immunology (IPATIMUP), University of Porto, 4200-135 Porto, Portugal; 7 CINTESIS@RISE (Health Research Network), 4200-319 Porto, Portugal; 8 Department of Pathology, Faculty of Medicine, University of Porto, 4200-319 Porto, Portugal

**Keywords:** serous effusion cytology, BlueStain, Papanicolaou stain, Giemsa stain, International System for Reporting Serous Fluid Cytopathology, cytopathological staining

## Abstract

Serous effusion cytology is a pivotal diagnostic and staging tool in clinical pathology, valued for its simplicity and cost-effectiveness. Staining techniques such as Giemsa and Papanicolaou are foundational, yet the search for rapid and efficient alternatives continues. Our study assesses the efficacy of an in-house-developed BlueStain, a toluidine blue variant, within the International System for Reporting Serous Fluid Cytopathology (TIS), aiming to optimize diagnostic clarity and resource use. Materials and Methods: This section provides details on the cohort of 237 patients with serous effusions, the ethical approval process, sample collection, and staining procedures with BlueStain, Papanicolaou, and Giemsa. It also describes the microscopic evaluation criteria, scoring system, and statistical methods used to compare the stains. Results: BlueStain demonstrated notable performance, particularly in identifying malignant cells, presenting a competitive alternative to the Papanicolaou stain, which, despite higher quality indices in other categories, requires more resources and time. The study revealed that BlueStain might offer a valuable balance between quality and efficiency, especially in cases where rapid diagnostic turnaround is essential. Conclusions: Our findings suggest that BlueStain is a viable staining method in the context of serous effusions, capable of providing detailed cytomorphological analysis. While traditional stains hold their place for their established diagnostic clarity, BlueStain offers a rapid and resource-optimized alternative. The absence of definitive diagnostic criteria in the atypical category and the inherent sample heterogeneity underscores the necessity for adaptable staining methods like BlueStain. The study highlights the potential trade-offs between detail and practicality in staining techniques, advocating for further research into innovative methods that do not compromise diagnostic precision for cost and time efficiency.

## 1. Introduction

Cytological sampling from body cavities has long been recognized as an essential tool in both diagnostic and staging evaluations of serous effusions [1,2,3,4,5]. Its primary advantages include simplicity, safety, and cost-effectiveness, and it has solidified this reputation through widespread clinical use [1,2,3,4,5]. The data extracted from these samples vary between laboratories due to different collection and preparation techniques, including the use of different stains, and varying levels of experience among cytopathologists. Standardized reporting systems have effectively addressed this issue in several areas of cytopathology [1,6,7,8].

In cytopathology, the choice of an appropriate stain is crucial for an accurate diagnosis. Commonly used cytology stains include Giemsa-based stains and the Papanicolaou (Pap) stain, each offering unique insights into cellular morphology and background elements [2,3,4,9,10].

Biological stains play a pivotal role in cytopathology, serving as indispensable tools to enhance the visualization and interpretation of cellular structures and components. These stains are specifically designed to selectively bind to different cellular elements, such as nuclei, cytoplasm, and specific organelles, thereby highlighting their morphology and aiding in the identification of pathological changes. By imparting distinct colors or contrasts to cellular features, stains enable pathologists to discern normal from abnormal cells, identify specific cell types, and detect abnormalities indicative of disease processes. Overall, the primary purpose of biological stains in cytopathology is to facilitate accurate and comprehensive cytological evaluations, ultimately contributing to the diagnosis, prognosis, and management of various medical conditions.

Our research group has previously assessed the utility of an original stain, BlueStain, developed in-house for use in rapid on-site evaluation (ROSE) and tested for thyroid fine needle aspiration biopsy (FNAB) samples, employing the Bethesda reporting system [10]. This stain, a toluidine blue variant, has shown promising results in providing rapid and reliable assessments of specimen adequacy and preliminary diagnoses.

BlueStain is an acid solution for metachromatic cell staining with 1% toluidine blue (Toluidine Blue O), certified by the Biological Stain Commission, soluble in ethyl alcohol 70%. This in vitro diagnostic (IVD) medical device for cytoplasmic and nuclear staining serves two primary purposes: defining nuclear details, facilitating a precise analysis of inflammatory and neoplastic alterations, and ensuring cytoplasmic transparency, which is particularly important due to varying cell thicknesses and the frequent overlap of cells in the sample. Toluidine blue (TB) generally uses various solvents in addition to water such as acetic acid and other types of alcohol. Its staining protocol varies depending on the type of biological sample, and it can take several minutes to complete [11]. In contrast, BlueStain has just two steps: staining the slides for 90 s and rinsing in tap water. BlueStain dye’s ability to effectively stain various cytological sample types, including both conventional and ThinPrep smears, and its compatibility with samples fixed in alcohol or air-dried at room temperature, position it as a valuable alternative staining dye for cytology labs. This versatility not only simplifies staining procedures but also ensures consistent and reliable results, enhancing the efficiency and accuracy of diagnostic processes in the lab.

Building on this foundation, our current study aims to extend the application of the original BlueStain for final diagnostic evaluation in serous effusions, exploring its efficacy within the framework of the International System for Reporting Serous Fluid Cytopathology (TIS) [1]. This system is designed to standardize the reporting of serous fluid cytology by categorizing findings into distinct diagnostic groups, enhancing clinical decision-making and patient management. The TIS system has proven instrumental in reducing inadequacy rates, minimizing the need for repeat studies and improving overall healthcare resource utilization [1,2,3].

The value of a precise and effective staining method in this context cannot be ignored. The role of stains in cytopathology extends beyond mere coloration; they are integral in highlighting critical cytological features, aiding in the accurate categorization of samples as per the TIS system [1]. Through this study, we aim to evaluate the potential efficacy of BlueStain in enhancing the diagnostic accuracy of serous effusion cytology. By comparing it with established staining methods like Giemsa and Pap, we intend to assess its relative efficacy in terms of clarity, reliability, and diagnostic utility. This research could potentially lead to more efficient and cost-effective practices in the analysis of serous effusions, ultimately contributing to the continuous evolution and improvement of cytopathological techniques.

## 2. Materials and Methods

### 2.1. Sample Collection and Processing

This research encompassed a cohort of 237 patients, each presenting with serous effusions, who were referred for evaluation. The study protocol received approval from the Committee for Ethical and Responsible Conduct of Research (N16/2022). A total of 237 cytological specimens were analyzed in this study, comprising 55 peritoneal lavage samples, 112 pleural effusion samples, and 70 ascites samples. These specimens were subjected to three different staining procedures: BlueStain, Pap stain, and Giemsa stain. However, not all samples were available for all three stains at the same time due to volume restrictions and different hospital practices. The distribution of stains across the samples was as follows: all 237 cases were available for BlueStain, 228 for Pap stain, and 118 for Giemsa stain.

### 2.2. The Application of the BlueStain Method

For this study, serous effusion fluid was collected from each patient and placed in a suitable container. Depending on the specific protocol of the institution, the fluid was either centrifuged to obtain the sediment or processed using a Cytospin preparation to concentrate the cells. For bloody specimens, a red blood cell (RBC) lysis buffer based on ammonium chloride was added to selectively lyse RBCs without damaging other cell components. After incubation for 10 min at room temperature, we proceeded with centrifugation. Abscess-filled specimens that were very thick were diluted slightly with saline to improve the homogeneity. In these cases, the Cytospin parameters were adjusted based on the cell density and viscosity of the sample.

A smear was prepared from the sediment or concentrated cells on a slide, with a brief air-drying period if required. Priority in staining was given to our BlueStain technique [12]. Each prepared smear slide was dipped in BlueStain for 90 s and then gently rinsed by dipping in tap water 5–7 times. In cases where additional slides were available, they were stained using Papanicolaou and Giemsa stains according to the classical protocols [9,10,11,12,13]. This approach allowed for a comprehensive evaluation of the serous effusion samples, utilizing the advantages of each staining method to provide a thorough cytological analysis.

### 2.3. Cytological Microscopic Evaluation

The slides prepared from serous fluid samples were examined under a microscope by two experienced cytopathologists (S.C. and F.S.). The evaluation criteria included background, overall stain, cellularity, cell morphology, cytoplasmic details, nuclear characteristics, cellular pleomorphism, nuclear chromatin, and nucleoli. Each parameter was scored on a scale of 3, 2, or 1, corresponding to good, average, and poor quality, respectively. Consequently, nine scores were multiplied to produce a final score, and the maximum possible score for a specimen, considering all parameters, was 27.

The respective definitions for good, average, and poor quality for evaluating the slides under the microscope by the two cytopathologists were:

Good quality (score of 3):

- Background: The slide background is clear and free of artifacts or unwanted staining that could obscure cellular details.
- Overall stain: The staining is appropriate in intensity, enhancing the visibility of cellular structures without overpowering or under-representing any features.
- Cellularity: The specimen contains an ample number of cells with the staining uniformly distributed along the slide.
- Cell morphology: Cells maintain typical morphology with well-preserved structures, facilitating easy identification and classification.
- Cytoplasmic details: The cytoplasm of cells is clearly visible and shows distinct boundaries and internal structures where applicable.
- Nuclear characteristics: Nuclear contours are sharp; nuclei are appropriately sized and shaped, with any physiological variations clearly discernible.
- Cellular pleomorphism: Variation in cell size and shape is minimal, reflecting a homogeneous cell population or the expected heterogeneity in reactive or neoplastic conditions.
- Nuclear chromatin: The chromatin pattern is distinct and evenly distributed, indicating good preservation and staining.
- Nucleoli: If present, they are clearly visible and well-defined, appropriate for the cell type and pathological state.

Average quality (score of 2):

- Background: The slide background shows minor artifacts or staining issues, but these do not significantly hinder the observation of cellular features.
- Overall stain: Staining is generally adequate though may be uneven in some areas, with some cellular features less clearly highlighted.
- Cellularity: The cell density is moderate, with the staining not completely uniformly distributed along the slide.
- Cell morphology: Some cell structures are slightly distorted or less distinctly preserved, posing minor challenges to identification.
- Cytoplasmic details: Cytoplasmic boundaries and contents are visible but may lack sharpness or detail.
- Nuclear characteristics: Nuclear details are reasonably clear; however, slight blurring or irregularities in size and shape may be present.
- Cellular pleomorphism: There is noticeable variability in cell size and shape, which may slightly complicate the evaluation.
- Nuclear chromatin: Chromatin is visible but may show some clumping or irregular distribution.
- Nucleoli: Nucleoli are discernible but may lack crisp definition or uniform appearance.

Poor quality (score of 1):

- Background: The background is cluttered with artifacts or excessive staining, significantly obstructing cellular detail observation.
- Overall stain: Staining is poor, either too faint or overly intense, obscuring critical cellular features.
- Cellularity: The cell density is sparse or moderate, with the heterogeneous distribution of the staining along the slide.
- Cell morphology: Cell structures are poorly preserved or markedly distorted, making reliable identification challenging.
- Cytoplasmic details: Cytoplasmic features are blurred or indistinguishable, with poor definition of internal and boundary structures.
- Nuclear characteristics: Nuclei are poorly defined, overstained and without clear details on chromatin and nucleoli.
- Cellular pleomorphism: High variability in cell size and shape is present, exceeding expected norms and complicating analysis.
- Nuclear chromatin: Chromatin appears smeared, overly clumped, or unevenly distributed, detracting from diagnostic accuracy.
- Nucleoli: Nucleoli, if visible, are poorly defined or irregular, providing little diagnostic value.

**Figure 1:**
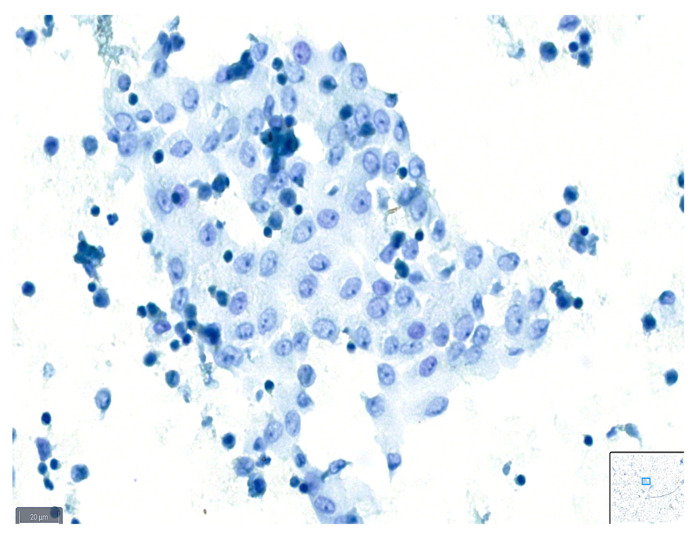
Example of a case classified as good quality using BlueStain. See the clear nuclei and cytoplasm details. Peritoneal effusion: benign, reactive mesothelial cells. (BlueStain; original magnification 40×).

**Figure 2:**
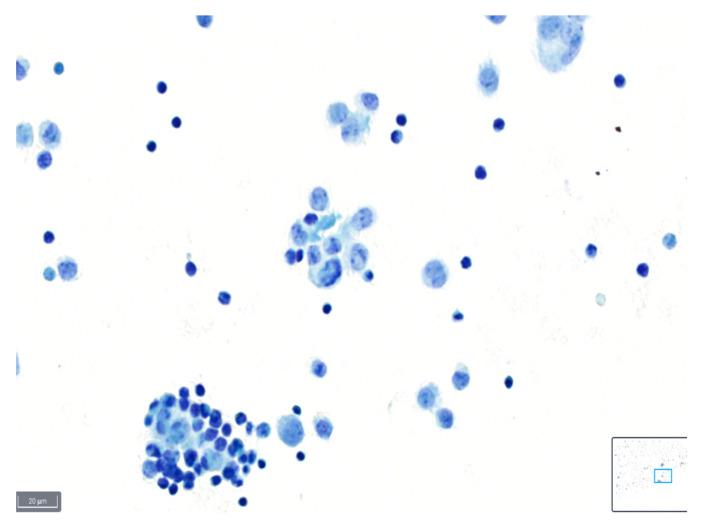
Example of a case classified as average quality using BlueStain. Nuclear details are somewhat clear; however, slight blurring is present. Pleural effusion: malignant, metastatic lung adenocarcinoma. (BlueStain; original magnification 40×).

**Figure 3:**
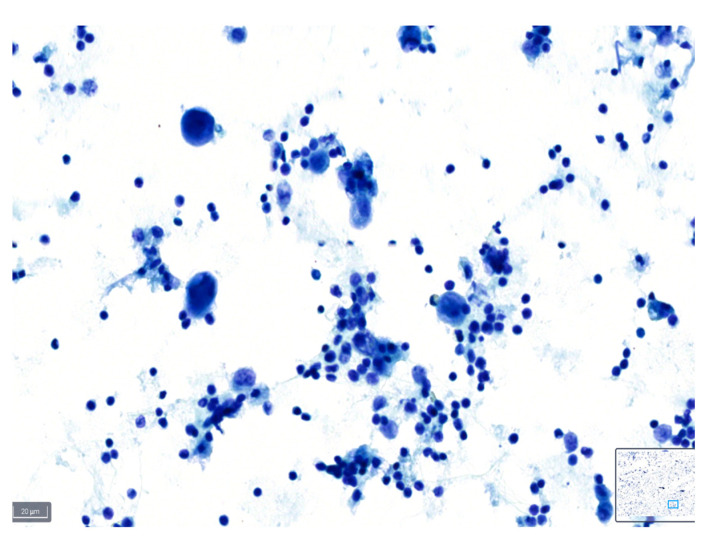
Example of a case classified as poor quality using BlueStain. Nuclei are overstained and without clear details on chromatin and nucleoli. Pleural effusion: malignant, metastatic lung adenocarcinoma. (BlueStain; original magnification 40×).

These definitions can serve as a guideline for cytopathologists when assessing the quality of slides based on the various parameters described, and examples are shown in Figure 1, Figure 2 and Figure 3.

Each case was evaluated based on TIS, which classifies cytology reports into distinct categories [1]:

ND (non-diagnostic): samples with an insufficient number of cells for cytologic interpretation, without any atypia.

NFM (negative for malignancy): samples with cellular changes that completely lack evidence of mesothelial or non-mesothelial malignancy.

AUS (atypical cells of undetermined significance): samples that lack enough quantitative or qualitative cytologic features to be confidently diagnosed as either benign or malignant, and representing a true gray zone in effusion cytology.

SFM (suspicious for malignancy): samples showing cytologic features usually present in malignant lesions but insufficient in quality or quantity for a definitive diagnosis of malignancy. The type of malignancy suspected (epithelial, mesothelial, lymphoid, other) should always be stated.

MAL (malignant): samples showing cytomorphologic features that, either alone or combined with the results from ancillary studies, are diagnostic of a primary or secondary malignancy.

### 2.4. Quality Index Calculation

The quality index (QI) for each case was calculated by dividing the maximum possible score (=27) by the actual score obtained for that case. This index aimed to provide a standardized measure of the cytological specimen quality [12].

### 2.5. Resolution of Discordant Scores

In cases where the initial evaluations by the two pathologists (S.C. and F.S.) resulted in discordant scores, a consensus was reached through joint review using a multi-head microscope. The agreed-upon score was then used for the final analysis.

### 2.6. Statistical Analysis

Statistical analysis was performed using IBM SPSS Statistics 29.0 (IBM, New York, NY, USA) and graphical construction was carried out using GraphPad Prism 8 software version 8.0.2 (GraphPad, San Diego, CA, USA). Outlier analysis was carried out, and only extreme outliers were excluded. The Kolmogorov–Smirnov and Shapiro–Wilk tests were used to assess the distribution of the data. The Kruskal–Wallis test with the Bonferroni correction was used for multiple comparisons.

## 3. Results

**Figure 4:**
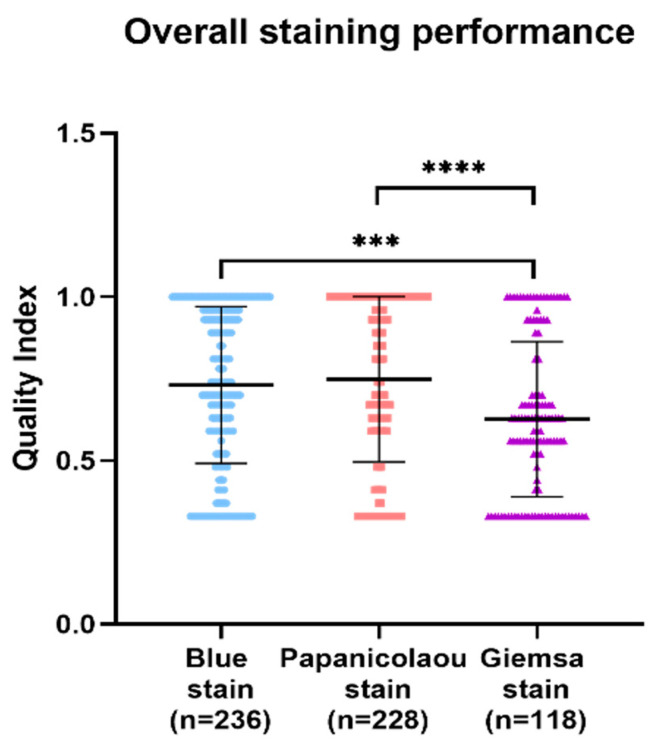
Overall analysis of BlueStain, Papanicolaou, and Giemsa performance. Results are shown as median ± IQR. *** *p*-value ≤ 0.001 and **** *p-*value ≤ 0.0001.

In the comparative analysis of staining techniques for a series of cytology samples, the Papanicolaou stain demonstrated superior quality index results, followed by BlueStain and the Giemsa stain. Statistically significant differences were observed, with the Giemsa stain exhibiting a notably lower quality index compared to BlueStain and the Papanicolaou stain (Figure 4). The *p*-values for the overall comparisons were as follows: BlueStain vs. Papanicolaou (*p* = 0.9241), BlueStain vs. Giemsa (*p* = 0.0005), and Papanicolaou vs. Giemsa (<0.0001) (Appendix A Table A1).

**Figure 5:**
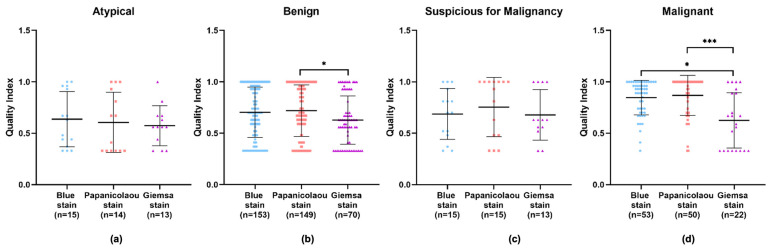
Performance of BlueStain, Papanicolaou, and Giemsa according to the categories (**a**) atypical, (**b**) benign, (**c**) suspicious for malignancy, and (**d**) malignant. The results are presented as median ± IQR. * *p*-value ≤ 0.05 and *** *p*-value ≤ 0.001.

An in-depth examination of the data across different diagnostic categories—benign, atypical, suspicious for malignancy, and malignant—revealed that the Giemsa stain consistently showed the lowest quality index in all categories. Apart from the atypical category, the Papanicolaou stain displayed higher quality indexes in the remaining categories. However, no significant difference can be observed in the diagnostic efficacy of the Papanicolaou stain compared to others within the atypical category, implying that while it may not be superior, it is also not inadequate (Figure 5a–d and Appendix A Table A2). Particularly in the benign and malignant categories, the Giemsa stain’s quality index was significantly lower compared to the Papanicolaou stain (Figure 5b,d), with *p*-values indicating strong statistical significance.

In the comparative evaluation of cytological staining techniques, BlueStain demonstrated a consistent performance across various categories. While not surpassing the Papanicolaou stain in quality indices, it exhibited a statistically significant improvement over the Giemsa stain. BlueStain, in particular, showed a marked increase in quality index within the malignant category, suggesting its potential utility for precise diagnostic purposes in specific pathological contexts.

**Figure 6:**
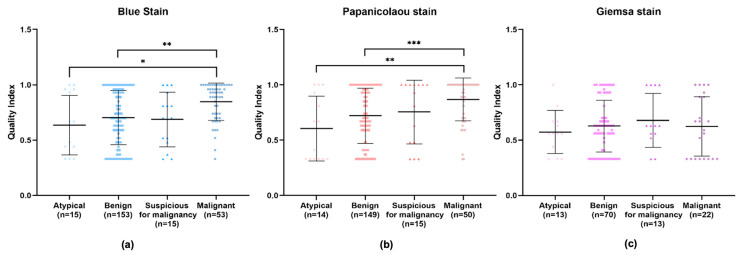
Individual performance of (**a**) BlueStain, (**b**) Papanicolaou, and (**c**) Giemsa. Results are shown as median ± IQR. * *p*-value ≤ 0.05, ** *p*-value ≤ 0.01, and *** *p*-value ≤ 0.001.

Upon an isolated analysis of each stain, the benign, suspicious for malignancy, and malignant categories demonstrated higher quality indexes compared to the atypical category (Figure 6). Specifically, BlueStain revealed a statistically significantly higher quality index for the malignant category over the benign and atypical categories (Figure 6a and Appendix A Table A3). A similar pattern was observed with the Papanicolaou stain (Figure 6b and Appendix A Table A3). Although no statistically significant differences were noted within the categories for the Giemsa stain (Figure 6c), it is noteworthy that the suspicious for malignancy category scored the highest quality index.

**Figure 7:**
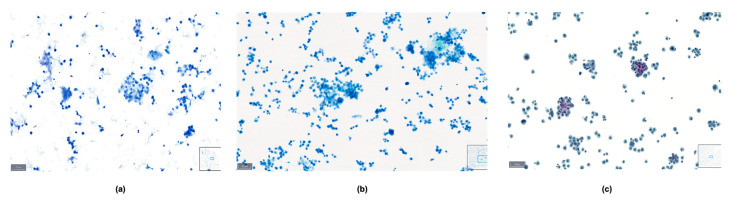
Comparison between (**a**) BlueStain, (**b**) Giemsa, and (**c**) Papanicolaou in a case of malignant pleural effusion.

The comprehensive scoring data for the quality index, which include parameters such as background, overall stain, cellularity, cell morphology, cytoplasmic details, nuclear characteristics, cellular pleomorphism, nuclear chromatin, and nucleoli, are detailed in Table 1. The Papanicolaou stain outperformed the other stains in most parameters, with BlueStain following closely. The Giemsa stain generally showed a lower performance across these criteria. The comparison of the three staining methods in a malignant pleural fluid sample is presented in Figure 7.

**Figure 8:**
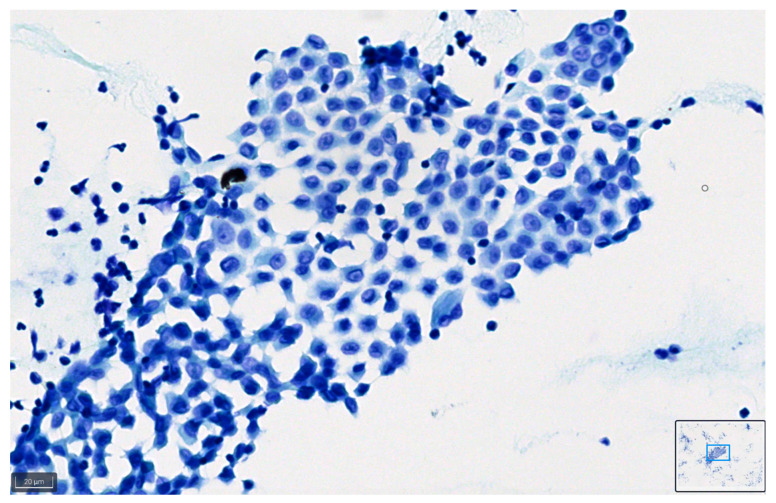
Pleural effusion: benign mesothelial cells stained with BlueStain. Note the good preservation of nuclei and cytoplasm characteristics of the cells (BlueStain; original magnification 40×).

**Figure 9:**
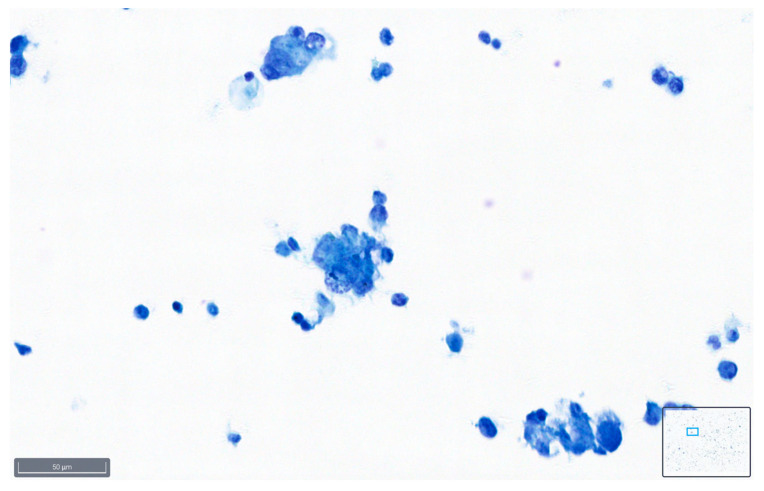
Peritoneal effusion. Groups of malignant cells from a metastatic colon carcinoma. Note the preservation of the malignant characteristics of the cells as coarse chromatin in the blue staining (BlueStain; original magnification 40×).

Figure 8 and Figure 9 show representative images of benign and malignant cells in effusions, stained with BlueStain. We can observe the nuclei and cytoplasmic details that allow for a correct diagnosis/classification of the case.

## 4. Discussion

A cytological examination of serous fluids is commonly used to initially assess the cause of fluid accumulation in body cavities. This diagnostic procedure is minimally invasive, easily accessible, and cost-effective. In cases of malignant effusion, it can be used to identify the source of cancer when serous fluid accumulation is the first symptom. Additionally, it aids in determining cancer stage, recurrence, and prognosis. However, the reported diagnostic accuracy varies widely due to factors such as collection and preparation methods, similarities in appearance between benign and malignant cells, and the expertise of the pathologist interpreting the results [1,2,3,4,5,14].

Optimal specimen preparation is crucial for accurate diagnostic interpretation. Various preparation methods are utilized, including conventional direct smears, cytocentrifugation preparations, liquid-based preparations, and cell blocks. Ancillary tests can be conducted on most of these preparations. Standard staining techniques involve Papanicolaou stains for alcohol-fixed smears and preparations, modified Giemsa stains for air-dried smears and preparations, and hematoxylin and eosin (H&E) staining for formalin-fixed, paraffin-embedded (FFPE) specimens. Employing multiple preparation methods and stains for one specimen enhances the amount of information available, aiding in interpretation.

Recently, a task force appointed by the International Academy of Cytology and the American Society of Cytopathology published the results of a web-based survey with 54 questions to identify opinions and explore existing practice patterns regarding body fluid cytopathology [14]. Most (72.9%; 291/399) respondents of the survey believed that alcohol fixation should be used whenever possible; however, 74% (295/401) agreed that both the Papanicolaou stain and modified Giemsa stain (e.g., Diff-Quik) should be used as standard preparations in serous fluid specimens whenever possible [14].

In the evolving landscape of cytopathological staining methods, our study’s exploration of BlueStain in serous effusions aligns with the broader quest for rapid, cost-effective, and highly detailed staining techniques. Traditional methods like Papanicolaou and Giemsa stains, despite their diagnostic clarity and established utility, pose challenges in terms of time and resource requirements [9,12,13,15]. This has catalyzed the exploration of alternative staining methods, as evidenced by the research conducted by Amannagi et al. [16] and Hewer et al. [17] and our own previous investigation [12].

The work by Amannagi et al. [16] explored the utility of toluidine blue in the rapid on-site evaluation of thyroid cytological smears. Their findings underscore the importance of rapid staining techniques, highlighting how such methods can facilitate prompt preliminary assessments without compromising diagnostic accuracy. Similarly, Hewer et al. [17] also investigated toluidine blue in thyroid FNAB, emphasizing its efficiency in cytology reporting. However, similar to Amannagi’s study [16], Hewer et al. [17] concentrated primarily on sample adequacy rather than a comprehensive cytological evaluation. Few studies have assessed the quality of staining beyond the efficiency of rapid on-site evaluation, prioritizing speed over staining precision in cytological assessments. Deepthi et al. [9] employed a quality index score comparable to that utilized in our investigation to evaluate the effectiveness of a modified ultrafast Giemsa stain in FNAC across various organs, demonstrating the viability of this approach in routine cytological practice.

Rapid on-site evaluation is still underused in cavity fluids and can be useful in some situations, for example to avoid a second pleuroscopy procedure for talc poudrage in malignant effusions, to predict malignant pleural effusion during diagnostic pleuroscopy [18,19], and to select material for appropriate ancillary techniques [20].

Our previous study, which introduced the BlueStain method in thyroid FNAB samples using the Bethesda System for Reporting Thyroid Cytopathology, marked a significant advancement in this direction [12]. We demonstrated that BlueStain could deliver high-quality results, combining the much-needed aspects of lower costs and reduced preparation times. Building on these findings, our current study extends the application of BlueStain to serous effusions, a diagnostically more challenging sample type compared to thyroid FNAB. Our comprehensive analysis included detailed evaluation of a range of cytomorphological components, thereby providing a deeper understanding of BlueStain diagnostic capabilities.

In our analysis, BlueStain exhibited a commendable performance, especially in the malignant category, when closely compared with the Papanicolaou stain. This finding is critical, given that the Papanicolaou stain showed the highest quality index, except for the atypical category, and it is known that this stain demands more resources and an extended processing time [15,21].

The absence of clear diagnostic criteria for the atypical category, as opposed to the well-defined criteria for benign and malignant classifications, and its position as a grey zone category could explain why the Papanicolaou stain does not show enhanced efficacy in this group [1].

In contrast, BlueStain appears to provide a balance of quality and efficiency, emerging as a potentially advantageous option in this setting. Notably, the Giemsa stain, while renowned for its detailed cellular visualization, fell short in our study, indicating a potential trade-off between detail and practicality in certain staining methods. Additionally, the staining time of the three methods used was quite different. While the BlueStain method required only 2 min from specimen processing to microscopic observation, the Papanicolaou and Giemsa methods needed 29 and 32 min, respectively.

Another potential advantage of BlueStain is to minimize the environmental impact associated with the production and disposal of diagnostic material. Papanicolaou and Giemsa staining methods require significant amounts of reagents, including highly toxic substances like alcohol or xylene. While modifications to the Papanicolaou staining process have been suggested in the past to minimize environmental impact, these alternative methods have not been widely embraced by laboratories [21,22,23,24]. The BlueStain method significantly diminishes the environmental impact, slashing costs by up to 90% through the elimination of harmful reagents and the decreased reliance on bulky equipment and logistics. Additionally, it enables diagnoses with substantially lower energy consumption. Since BlueStain requires minimal equipment, there is a lower dependency on electrical equipment beyond basic laboratory lights and possibly a slide dryer. For Papanicolaou, it is necessary to use more laboratory equipment, such as automatic stainers, which are often electrically powered, and fume hoods due to the use of xylene and alcohols in the clearing and dehydration steps [22,23]. This results in higher energy consumption. Additionally, the environmental and safety management of simpler chemicals and reagents in BlueStain contributes to its lower energy profile [12,13,14,15,16].

A comparative look at the methodologies, sample types, and outcomes of these studies reveals insightful contrasts and commonalities, as summarized in Table 2. While Amannagi et al. [16] and Hewer et al. [17], as well our previous study [12], focused on thyroid FNAB samples, the present study ventured into the realm of serous effusions, adding a layer of complexity due to the inherent heterogeneity of these samples. Furthermore, the diagnostic categories assessed in our study, facilitated by the TIS system, allowed for a more detailed understanding of stain performance across a spectrum of cytological presentations. Moreover, the comprehensive evaluation of diagnostic categories enabled by the TIS system in our investigation serves as a crucial foundation for the future integration of BlueStain in routine clinical specimens. This approach provides a nuanced understanding of stain performance across diverse cytological presentations, essential for optimizing its utility in clinical practice. By meticulously analyzing the staining outcomes within distinct diagnostic categories, we gain insights into the strengths and limitations of BlueStain across various pathological contexts. This holistic assessment not only enhances our current understanding but also guides the strategic implementation of BlueStain as a valuable tool in diagnostic cytopathology.

In conclusion, our study, alongside the contributions of Amannagi et al. [16] and Hewer et al. [17], underscores the ongoing need for innovative cytological stains that offer diagnostic precision without the constraints of high costs and extended processing times. BlueStain emerges as a promising candidate in this regard, particularly in settings where rapid turnaround and resource optimization are critical. Future research should aim to expand the application of BlueStain across various cytological contexts and explore its integration with emerging digital pathology tools to further revolutionize cytological diagnostics [24].

This study not only reinforces the potential of BlueStain as a versatile and cost-effective stain for cytopathological evaluation but also opens new avenues for its application in more complex cytological analyses. Future research should aim to expand the application of BlueStain across various cytological contexts and explore its integration with emerging digital pathology tools, potentially revolutionizing cytological diagnostics.

## Figures and Tables

**Table 1:** Distribution of the scores used to calculate the quality index: 3—good; 2—average; 1—poor.

		BlueStain	PAP Stain	Giemsa Stain
**Background**	1	48 (20.3%)	51 (22.4%)	32 (27.1%)
	2	73 (30.8%)	50 (21.9%)	54 (45.8%)
	3	116 (48.9%)	127 (55.7%)	32 (27.1%)
	**Total**	**237 (100.0%)**	**228 (100.0%)**	**118 (100.0%)**
**Overall stain**	1	45 (19.0%)	42 (18.4%)	32 (27.1%)
	2	87 (36.7%)	70 (30.7%)	55 (46.6%)
	3	105 (44.3%)	116 (50.9%)	31 (26.3%)
	**Total**	**237 (100.0%)**	**228 (100.0%)**	**118 (100.0%)**
**Cellularity**	1	88 (37.3%)	65 (28.5%)	56 (47.5%)
	2	49 (20.8%)	42 (18.4%)	35 (29.7%)
	3	99 (41.9%)	121 (53.1%)	27 (22.9%)
	**Total**	**236 (100.0%)**	**228 (100.0%)**	**118 (100.0%)**
**Cell morphology**	1	44 (18.6%)	48 (21.1%)	33 (28.0%)
	2	76 (32.1%)	59 (25.9%)	55 (46.6%)
	3	117 (49.4%)	121 (53.1%)	30 (25.4%)
	**Total**	**237 (100.0%)**	**228 (100.0%)**	**118 (100.0%)**
**Cytoplasmic details**	1	59 (24.9%)	65 (28.5%)	42 (35.6%)
	2	71 (30.0%)	57 (25.0%)	46 (39.0%)
	3	107 (45.1%)	106 (46.5%)	30 (25.4%)
	**Total**	**237 (100.0%)**	**228 (100.0%)**	**118 (100.0%)**
**Nuclear characteristics**	1	64 (27.0%)	60 (26.3%)	51 (43.2%)
	2	77 (32.5%)	63 (27.6%)	38 (32.2%)
	3	96 (40.5%)	105 (46.1%)	29 (24.6%)
	**Total**	**237 (100.0%)**	**228 (100.0%)**	**118 (100.0%)**
**Cellular pleomorphism**	1	49 (20.7%)	50 (21.9%)	35 (29.7%)
	2	57 (24.1%)	57 (25.0%)	50 (42.4%)
	3	131 (55.3%)	121 (53.1%)	33 (28.0%)
	**Total**	**237 (100.0%)**	**228 (100.0%)**	**118 (100.0%)**
**Nuclear chromatin**	1	67 (28.3%)	62 (27.2%)	53 (44.9%)
	2	83 (35.0%)	64 (28.1%)	40 (33.9%)
	3	87 (36.7%)	102 (44.7%)	25 (21.2%)
	**Total**	**237 (100.0%)**	**228 (100.0%)**	**118 (100.0%)**
**Nucleoli**	1	71 (30.0%)	61 (26.8%)	56 (47.9%)
	2	76 (32.1%)	70 (30.7%)	34 (29.1%)
	3	90 (38.0%)	97 (42.5%)	27 (23.1%)
	**Total**	**237 (100.0%)**	**228 (100.0%)**	**117 (100.0%)**

**Table 2:** Advantages and disadvantages of BlueStain in the present and previous study as compared with other routine stains.

Stain	Advantages	Disadvantages	Study Focus
BlueStain (Current Study)	Cost-effective, rapid, effective in serous effusions	Slightly lower QI than Papanicolaou in some categories	Serous effusions using TIS system
BlueStain [12] (Previous Study)	Lower cost, high performance in ROSE, quick preparation	Focused on thyroid FNAB samples using TBSRTC 2nd edition	Thyroid FNAB samples
Toluidine Blue [16] (Amannagi et al.)	Rapid application, effective for ROSE in thyroid FNAB	Limited evaluation of cytomorphological features	Thyroid FNAB samples
Toluidine Blue [17] (Hewer et al.)	Efficient in cytology reporting, quick preliminary assessments	Primarily focused on sample adequacy	Thyroid FNAB samples
Giemsa	Detailed cellular visualization	Longer staining time, lower QI in our serous effusion study	Commonly used in cytopathology
Papanicolaou	Highest QI, excellent detail in cellular morphology	Resource-intensive, longer processing time	Widely used in cytopathology

## Data Availability

All data generated or analyzed during this study are included in this article. Further inquiries can be directed to the corresponding author.

## Appendix A

**Table A1:** *p*-Values for all the staining comparisons.

Test	*p*-Value
BlueStain vs. Papanicolaou stain	0.9241
BlueStain vs. Giemsa stain	**0.0005**
Papanicolaou stain vs. Giemsa stain	**<0.0001**

**Table A2:** *p*-Values for all the staining comparisons according to the diagnostic categories.

Test	*p*-Value			
	Atypical	Benign	Suspicious for Malignancy	Malignant
BlueStain vs. Papanicolaou stain	1.0000	1.0000	1.0000	0.3651
BlueStain vs. Giemsa stain	1.0000	0.0512	1.0000	**0.0101**
Papanicolaou stain vs. Giemsa stain	1.0000	0.0178	1.0000	**0.0001**

**Table A3:** *p*-Values of each stain according to the diagnostic categories.

Test	*p*-Value		
	Blue Stain	Papanicolaou Stain	Giemsa Stain
Atypical vs. Benign	1.0000	1.0000	1.0000
Atypical vs. Suspicious for Malignancy	1.0000	0.6751	1.0000
Atypical vs. Malignant	**0.0367**	**0.0056**	1.0000
Benign vs. Suspicious for Malignancy	1.0000	1.0000	1.0000
Benign vs. Malignant	**0.0039**	**0.0005**	1.0000
Suspicious for Malignancy vs. Malignant	0.2584	0.9834	1.0000

## References

[1] Pinto D., Chandra A., Crothers B., Kurtycz D., Schmitt F. (2020). The international system for reporting serous fluid cytopathology—Diagnostic categories and clinical management. J. Am. Soc. Cytopathol..

[2] Lobo C., Costa J., Petronilho S., Monteiro P., Leça L., Schmitt F. (2021). Cytohistological correlation in serous effusions using the newly proposed International System for Reporting Serous Fluid Cytopathology: Experience of an oncological center. Diagn. Cytopathol..

[3] Sachan R., Gupta A., Awasthi P.N., Singh P., Anand N., Chandra S., Gaur G., Husain N., Sachan K.D. (2023). Application of international system for reporting serous fluid cytology (ISRSFC) in effusion samples—A prospective study in an oncology setting. J. Am. Soc. Cytopathol..

[4] Altman E., Spiridonov L., Vadim S., Alejandro L., Alexei G., Hector C.I. (2013). Efficacy of cytology, cell blocks and thoracoscopic pleural biopsy in malignant pleural effusion diagnosis. Eur. Respir. J..

[5] Lu C., Liu C., Jhuang J.Y., Chen C. (2024). Comprehensive evaluation of benign and malignant etiologies of different serous effusions with the International System for Reporting Serous Fluid Cytopathology: A multi-institutional study in Taiwan. Cancer Cytopathol..

[6] Schmitt F., Bubendorf L., Canberk S., Chandra A., Cree I., Engels M., Hiroshima K., Jain D., Kholová I., Layfield L. (2023). The World Health Organization Reporting, System for Lung Cytopathology. Acta Cytol..

[7] Ali S.Z., Baloch Z.W., Cochand-Priollet B., Schmitt F.C., Vielh P., VanderLaan P.A. (2023). The 2023 Bethesda System for Reporting Thyroid Cytopathology. J. Am. Soc. Cytopathol..

[8] Nayar R., Wilbur D. (2015). The Bethesda System for Reporting Cervical Cytology: Definitions, Criteria, and Explanatory Notes.

[9] Deepthi B., Prayaga A.K., Rukmangadha N. (2022). Comparison of modified ultrafast giemsa stain with the standard may grunwald giemsa stain in FNAC of various organs. J. Cytol..

[10] Paessler M., LiVolsi V.A., Baloch Z.W. (2001). Role of ultrafast papanicolaou-stained scrape preparations as an adjunct to frozen sections in the surgical management of thyroid lesions. Endocr. Pract..

[11] Vidal B., Mello M.L. (2019). Toluidine blue staining for Cell and tissue biology applications. Acta Histochem..

[12] Alves P.M., Ferreira F., Oliveira T., Alves D., Canberk S., Schmitt F.C. (2023). A New Cytology Staining Method: A Fast Approach for Rapid On-Site Evaluation on Thyroid Fine-Needle Aspiration Cytology. Acta Cytol..

[13] Chantziantoniou N., Donnelly A.D., Mukherjee M., Boon M., Austin R.M. (2017). Inception and Development of the Papanicolaou Stain Method. Acta Cytol..

[14] VandenBussche C., Crothers B., Chandra A., Schmitt F., Kurtycz D. (2023). The International System for Reporting Serous Fluid Cytopathology: The Initial Project Survey. Cytopathology.

[15] Thakur M., Guttikonda V.R. (2017). Modified ultrafast Papanicolaou staining technique: A comparative study. J. Cytol..

[16] Ammanagi A.S., Dombale V.D., Patil S.S. (2012). On-site toluidine blue staining and screening improves efficiency of fine-needle aspiration cytology reporting. Acta Cytol..

[17] Hewer E., Schmitt A.M. (2020). Ultrafast Toluidine Blue Staining for Rapid On-Site Evaluation of Cytological Smears. Acta Cytol..

[18] Grosu H.B., Kern R., Maldonado F., Casal R., Andersen C.R., Li L., Eapen G., Ost D., Jimenez C., Frangopoulos F. (2022). Predicting malignant pleural effusion during diagnostic pleuroscopy with biopsy: A prospective multicentre study. Respirology.

[19] Wang H., Liu Y., Wang J., Ren T., Luo G., You H., Wang X., Li D., Wang L., Wang M. (2023). Rapid on-site evaluation of touch imprints of medical thoracoscopy biopsy tissue for the management of pleural disease. Front. Med..

[20] Pinto D., Schmitt F. (2020). Current applications of molecular testing on body cavity fluids. Diagn. Cytopathol..

[21] Priyanjali A., Toi P.C., Subramaniam H., Lakshmi S. (2019). Prospective comparison of cytological specimen adequacy assessment by different rapid staining techniques for rapid on-site evaluation in fine needle aspiration cytology and their cost-effectiveness. Diagn. Cytopathol..

[22] Moya-Salazar J., Rojas-Zumaran V. (2019). Eco-Pap: The Ecological Modification of the Papanicolaou Stain for Sustainable Cervical Cancer Diagnosis. Acta Cytol..

[23] Gupta S., Chachra K.L., Bhadola P., Sodhani P. (2010). Modified Papanicolaou staining protocol with minimum alcohol use: A cost-cutting measure for resource-limited settings. Cytopathology.

[24] Elmas H., Önal B., Steurer S., Hantzsch-Kuhn B., Claussen M., Mehdi E., Ince Ü., Rabe K.F., Sauter G., Welker L. (2023). Rapid Remote Online Evaluation in Endoscopic Diagnostics: An Analysis of Biopsy-Proven Respiratory Cytopathology. Diagnostics..

